# The HK2 Dependent “Warburg Effect” and Mitochondrial Oxidative Phosphorylation in Cancer: Targets for Effective Therapy with 3-Bromopyruvate

**DOI:** 10.3390/molecules21121730

**Published:** 2016-12-15

**Authors:** Paweł Lis, Mariusz Dyląg, Katarzyna Niedźwiecka, Young H. Ko, Peter L. Pedersen, Andre Goffeau, Stanisław Ułaszewski

**Affiliations:** 1 Institute of Genetics and Microbiology, University of Wroclaw, Przybyszewskiego Street, 51-148 Wroclaw, Poland; p.lis@dundee.ac.uk (P.L.); mariusz.dylag@uwr.edu.pl (M.D.); katarzyna.niedzwiecka@uwr.edu.pl (K.N.); 2 KoDiscovery, LLC, UM BioPark, Suite 502 E&F, 801 West Baltimore Street, Baltimore, MD 21201, USA; kocancer212@yahoo.com; 3 Departments of Biological Chemistry and Oncology and member at large Sidney Kimmel Comprehensive Cancer Center, Johns Hopkins University School of Medicine, Baltimore, MD 21205-2185, USA; ppederse@jhmi.edu; 4 Institut des Sciences de la Vie, Université Catholique de Louvain, B-1348 Louvain-la-Neuve, Belgium; agoffeau@hotmail.com

**Keywords:** 3-bromopyruvate, antitumor therapy, Warburg effect, Crabtree effect, oxidative phosphorylation, glutathione, buthionine sulphoximine

## Abstract

This review summarizes the current state of knowledge about the metabolism of cancer cells, especially with respect to the “Warburg” and “Crabtree” effects. This work also summarizes two key discoveries, one of which relates to hexokinase-2 (HK2), a major player in both the “Warburg effect” and cancer cell immortalization. The second discovery relates to the finding that cancer cells, unlike normal cells, derive as much as 60% of their ATP from glycolysis via the “Warburg effect”, and the remaining 40% is derived from mitochondrial oxidative phosphorylation. Also described are selected anticancer agents which generally act as strong energy blockers inside cancer cells. Among them, much attention has focused on 3-bromopyruvate (3BP). This small alkylating compound targets both the “Warburg effect”, i.e., elevated glycolysis even in the presence oxygen, as well as mitochondrial oxidative phosphorylation in cancer cells. Normal cells remain unharmed. 3BP rapidly kills cancer cells growing in tissue culture, eradicates tumors in animals, and prevents metastasis. In addition, properly formulated 3BP shows promise also as an effective anti-liver cancer agent in humans and is effective also toward cancers known as “multiple myeloma”. Finally, 3BP has been shown to significantly extend the life of a human patient for which no other options were available. Thus, it can be stated that 3BP is a very promising new anti-cancer agent in the process of undergoing clinical development.

## 1. Introduction

The “Warburg Effect” is cancers’ oldest known hallmark, i.e., aberrant sugar (glucose) metabolism to lactic acid even in the presence of oxygen. The renowned scientist Otto Warburg working in Berlin, Germany during the early part of the past century made two key experimental discoveries as follows:

(1) Cells from most cancers, regardless of the tissue of origin, exhibit a very intense sugar (glucose) consumption which may exceed 10-fold that of normal cells [1].
(2) This results in an elevated production and extrusion of lactic acid even in the presence of oxygen [1].

The net result of these two observations, i.e., “high level of glycolysis in cancer cells even in the presence of oxygen”, is now commonly and appropriately referred to as the “Warburg effect”. Following the subsequent discovery 18 years later that mitochondria are the site of ATP synthesis by oxidative phosphorylation [2,3], Warburg assumed that the high aerobic glycolysis that he and colleagues had observed previously in many cancers resulted from the lowered capacity of mitochondria to utilize oxygen, and may be a putative cause of cancer [4]. Although neither assumption was completely correct nor incorrect, there was much more to be learned as will be noted below as this brief review develops.

## 2. Experimental Knowledge Acquired to Date

### 2.1. Energy Production Considerations in Normal Cells and Cancer Cells

In most healthy cells, about 90% of the ATP is derived from mitochondrial oxidative phosphorylation, and only about 10% comes from the metabolism of glucose to pyruvic acid. However, in rapidly growing cancer cells derived from established tumors, the “Warburg effect”, i.e., high glycolysis even in the presence of oxygen, may provide up to 60% of the ATP with the remaining (~40%) being derived from mitochondrial oxidative phosphorylation [5]. Although these percentages are dependent on growth rate and may differ widely among different types of cancers, it is important to note that both glycolysis and mitochondria via ATP production are the two vital contributors to powering cancer growth and metastasis. A very common misconception among those unfamiliar with cancer metabolism is that most cancerous tumors thrive exclusively off the sugar glucose via glycolysis for their energy source. In fact, as noted above, most frequently they derive significant amounts of their energy in the form of ATP from both the “Warburg effect”, as well as from mitochondrial oxidative phosphorylation.

As stated above, it should be appreciated that the “Warburg effect” allows cancerous tumors and the viscous cells that comprise them to adapt to hypoxic conditions which frequently develop during their growth [6]. Although uncommon, there are exceptions as some gliomas, hepatomas, and breast cancer cell lines are more dependent on mitochondrial oxidative phosphorylation for their energy source, i.e., ATP [7]. This can be explained by the presence of various bioenergetic phenotypes from the exclusively glycolytic to highly oxidative phosphorylation [8]. As reviewed much earlier by co-author Pedersen [9], these cells are usually slow growing. Moreover, Pedersen and coworkers [8] also have shown in vitro that the most rapidly growing hepatoma tumor is the 3924A and the slowest growing is the 9618A. The 7800 tumor has an intermediate growth rate. The 3924A tumor, i.e., the fastest growing, has the highest glycolytic rate. One year later, it was also confirmed that slowdown in growth of a tumor lowers the rate of glycolysis, and in turn, faster growth intensifies this process [10]. Apart from the “Warburg effect”, some cancers also exhibit a phenomenon of the so called “Crabtree effect”. It is short-term and reversible (unlike the “Warburg effect”) and involves the suppression of respiration and oxidative phosphorylation by a high concentration of glucose [11,12]. This may occur despite the presence of functional mitochondria [11]. There are several hypotheses to explain the case of inhibition of respiration and oxidative phosphorylation by a high concentration of glucose [12]. Most likely, in tumor cells this phenomenon is regulated by multifaceted mechanisms [7]. These include inter alia a competition for the free cytoplasmic phosphate pool (Pi) between the mitochondrial phosphate carrier and glyceraldehyde-3-phosphate dehydrogenase (GAPDH) or for free adenosidne diphosphate (ADP) between the mitochondrial adenine nucleotide translocase and the pyruvate kinase (PK). This emulation between glycolysis and oxidative phosphorylation can cause respiratory impairment by increased glucose availability. Furthermore, the important factor might be the action of fructose 1,6-biphosphate that inhibits the mitochondrial respiratory complexes III and IV. This leads to the inhibition of the ATP synthase. It should not be forgotten that the metabolism of glucose increases the reactive oxygen species (ROS) generation that damages the mitochondrial membrane and depresses respiration [7]. This phenomenon also was described for “Crabtree positive yeast’’, the cells of which were cultivated in media that contained a high concentration of glucose. This effect was especially well visible for cells during the logarithmic phase of growth. Inside the cells which undergo a “Crabtree effect”, respiration and stress response mechanisms are repressed. As a matter of fact, hexokinase 2 (HK2) is both highly elevated in rapidly growing cancers and is bound to mitochondrial voltage dependent anion channels (VDAC). When bound to VDAC, the HK2 is not inhibited by its product, i.e., glucose-6-phosphate (G-6-P). Therefore, glycolysis is elevated thus enhancing this pathway’s ATP production. Consequently, the need for ATP produced by oxidative phosphorylation, while still taking place to some extent, is suppressed/diminished.

These cells obtain energy mainly by fermentation of glucose. Depletion of glucose forces the cells to switch to the metabolism of non-fermentable carbon sources [13,14]. Furthermore, in some types of cancer, lactate released by the hypoxic tumor cell compartment can be transported to the aerobic tumor cell compartment which undergoes oxidative metabolism. Therefore lactate released by one cell drives the metabolism of the second cell. This phenomenon called “lactate shuttling” plays an important role for metabolic symbiosis establishment between glycolytic cells and cells that undergo oxidative metabolism [15,16]. Moreover, it has been shown many times that cancer cells can reversibly regulate their energy metabolism. This phenomenon observed in vitro must exist also in vivo. With respect to the tumor architecture, the metabolic symbiosis may be considered as a niche where a hypoxic central part utilizes glucose while an edge of the tumor which is better vascularized uses lactate as a substrate [16]. Therefore, some of the cancer cells within a tumor may have a different phenotype than the other cells [17]. Thus, the ability of cancer cells to undergo short-term and reversible changes in their metabolism may also allow them to avoid the toxic effect of a drug. This is possible as it has been shown in experiments performed in vitro [18,19] that chosen cancer cells are able to overcome the inhibition of fermentative metabolism. Thus, even very effective known inhibitors of glycolysis are not sufficient in eliminating cancer cells with changed metabolism. Therefore, the proper targeting of cancer cells remains an urgent problem [7].

On the other hand, as nutrients are usually in abundance, it has been suggested that cancer cells do not pursue the most energetically efficient biosynthetic pathways but rather the fastest ways necessary for rapid proliferation [20]. Not only is the sugar glucose vastly consumed by cancerous cells exhibiting the “Warburg effect” but also amino acids, mostly glutamine, which in addition to serving as an energy source [21] is also a source of amine groups essential for many biosynthetic events, e.g., production of purines and pyrimidines [22]. In addition, cancer cells may utilize metabolic pathways that are highly energy producing, e.g., the mitochondrial beta-oxidation of fatty acids [23]. Finally, it is important to note that the products and intermediates of glycolysis and glutaminolysis are used in the synthesis of many of the molecules needed for intense proliferation [20].

However, as noted much earlier [9], there is a marked reduction in mitochondria in many rapidly growing cancers, greatly diminishing their net contribution to the production of ATP essential to support cell growth and division. Therefore, in such cancers it is important to maintain a high glycolytic rate. The oxidized form of nicotinamide adenine dinucleotide (NAD^+^) and the nicotinamide adenine dinucleotide phosphate (NADPH_2_) are constantly needed both for the proper functioning of glycolytic enzymes and to maintain the high glycolytic flux. NAD^+^, which is necessary for the action of GAPDH is regenerated mainly by dehydrogenases, e.g., lactate dehydrogenase (LDH) which simultaneously converts pyruvate into lactate [20].

It was previously thought that pyruvate kinase M2 (PKM2), the embryonic form of this enzyme, is the form predominantly expressed in cancer cells. However, analyses of *PKM1* and *PKM2* gene expression in different cell lines did not confirm this [24]. Nevertheless, the activity of PKM is usually increased in cancer cells consistent with their upregulated glycolysis. PKM2 activity may be increased by its tetramerization and phosphorylation [25] rather than overexpression. It was also shown that the transition of PKM2 to PKM1 on the expression level diminishes the Warburg effect [15,26]. On the level of individual cells, the decrease of this cytosolic enzyme activity correlates with the activity of glycolytic enzymes, i.e., HK2 and GAPDH. Thus, this leads to decreasing of the lactate rate production in the presence of oxygen, what is very important for the functioning of tumor cells. Consequently, this phenomenon significantly reduces the growth rate of cancer cells [15,26,27,28].

A significant amount of pyruvate for LDH comes also from glutaminolysis and transamination of alanine [6]. The pyruvate dehydrogenase (PDH) in cancer cells exists in the phosphorylated inactive state. Therefore, most of the pyruvate becomes a substrate for LDH [29].

It has also been shown that a high lactate production induces overexpression of mono-carboxylate transporters (MCTs) in order to extrude excessive lactic acid and avoid cytoplasm acidification [30]. This leads to acidification of the tumor’s local environment resulting in degeneration of surrounding tissue and likely promotes invasion and metastasis [31]. Also, the extracellular lactate may be taken up, presumably also by MCTs, and utilized by other cancer cells possessing functional mitochondria [32,33]. Recently, the role of MCTs in human cancers has been reviewed [30].

Although much has been learned about the underlying molecular basis of the “Warburg effect” complete agreement on some aspects of this common cancer phenotype is lacking. Generally, it is assumed that the “Warburg effect” results from an increased glucose uptake together with glycolysis up-regulation and mitochondrial metabolism down-regulation [11,34]. It is often the case that substrate availability is the only limiting factor for a catalytic reaction and even a whole subsequent pathway. Therefore, it could be argued that an increased expression of glucose transporters (GLUTs) may be responsible, at least in part, for the “Warburg effect”. In fact, it was recently shown that the GLUT1 and GLUT3 transporters are overexpressed in numerous cancers [35]. However, it is also possible that the latter may be just one of the consequences of the “Warburg effect” [36]. Although it seems likely that the overexpression of one or more of the enzymatic participants involved in the glycolytic pathway, e.g., hexokinase (HK), phosphofructokinase (PFK), PK, and LDH contribute to the “Warburg effect”, it seems clear that mitochondrial bound hexokinase is essential [37,38]. In fact, it was shown in the latter study [38] that the addition of tumor mitochondria (from Ehrlich ascites cancer cells) containing bound hexokinase to liver cytosol lacking mitochondria and exhibiting no capacity to catalyze glycolysis, results in a glycolytic rate as high as that found in the cancer cells under study. Later the isoform involved was identified as HK2 [39]. It is now known that HK2 is highly expressed in most cancers whereas in most normal cells the predominant isoforms used are HK1, HK4, and to a lesser extent HK2 and HK3 [7].

In addition to being highly expressed in cancer cells and playing the major role in the “Warburg effect”, it is known also from an earlier collaborative project involving co-author Pedersen that the mitochondrial binding of hexokinase (now known to be HK2) is bound to the outer membrane protein VDAC [40]. This localization of HK2 gives it preferred access to ATP produced during oxidative phosphorylation [41], and unlike other hexokinase isoforms, causes it to become insensitive to inhibition by its product, G-6-P [42,43].

Abnormal expression of PFK was also shown in some cancer cell types. For example, leukemia and lymphoma cells express PFK isoforms which normally are exclusively expressed in liver and platelets. These isoenzymes, expressed in increased amounts, show a much lower sensitivity to inhibition and are easily activated even at low concentrations of 2,6-bisphosphate [44]. Other possible contributors to the Warburg effect may exist also at the level of pyruvate metabolism, i.e., its lowered transport into mitochondria, decreased activity of PDH and/or increased activity of LDH [7].

HIF-1α (hypoxia inducible factor-1 alpha) activation also is a contributor to the Warburg effect, likely at the transcriptional level. HIF-1α is usually activated as a consequence of mutations in tumor suppressor genes (e.g., P53, P21) [45] and contributes in part to the overexpression of glucose transporters and several glycolytic enzymes such as HK2, PFK, PKM, and LDH [46]. Perhaps most notable is the earlier finding that the HK2 promoter is activated seven-fold by hypoxic conditions in the presence of the first substrate of glycolysis, i.e., glucose [47]. Moreover, HIF-1α induces expression of pyruvate dehydrogenase kinase (PDK) [29], which inhibits PDH by its phosphorylation causing the vast majority of pyruvate to be converted into lactate by LDH. Expression of LDH itself is induced not only by HIF-1α but also through oncogenes (e.g., c-Myc) [20].

Collectively, in order to proliferate, tumors require large amount of energy in a short period of time. Consequently, aerobic glycolysis has an advantage as it provides ATP faster than mitochondrial respiration. All the above-mentioned metabolic changes render cancer cells less dependent on oxygen availability, support increased proliferation and migration, and help the cells avoid apoptosis and chemotherapy [6]. Although the consequences of the Warburg effect are clear, it is hard to unambiguously ascribe any one of the above-described molecular events as its only cause. Nevertheless, HK2 clearly stands out as the major contributor for the reasons already alluded to above. This and the fact that HK2 bound to mitochondrial VDAC [40] is also a major contributor to the immortalization of cancer cells [48,49] makes this isoenzyme one of cancer’s best friends. However, this also makes HK2 one of most vulnerable cancer’s “Achilles heel” and therefore it is a highly promising drug target. It is also worth mentioning that numerous mutations present in each tumor might come as a secondary side effect of metabolic changes related to the cancer disease. They arise after the initiation of uncontrolled growth due to damaged mitochondria [50].

### 2.2. Anti-Energy Metabolism Inhibitors: An Effective Approach for Treating Cancer

There is a very effective diagnostic technology based on mitochondrial bound HK2, the key player in the “Warburg effect”. Specifically, the technology referred to as positron emission tomography (PET) is based on the fact that in cancer cells 2-deoxyglucose (2-DG), a glucose analog, upon entering cancer cells is phosphorylated by mitochondrial bound hexokinase-2. This yields 2-DG-6-phosphate that is not further metabolized and thus accumulates in much higher levels than in normal cells. 2-DG used in PET is labeled with the ^18^fluorine radioisotope that decays, emitting a positron. This then annihilates with an electron giving two gamma-photons that are detected by a detector [45]. PET, based on mitochondrial bound HK2, is one of the most effective methods for imaging solid tumors and as such serves also as an effective method to screen for anticancer drugs.

2-DG, citrate, and 3-bromopyruvate (3BP) have all been tested recently as potential anticancer drugs acting either via glycolysis inhibition (2-DG and citrate) or glycolysis plus mitochondrial inhibition (3BP). It was shown in vitro that 7-days therapy with 5 mM 2-DG resulted in a strong but incomplete inhibition of growth (60% to over 90%) in 12 different cancer cell lines [51]. A dramatic 100% mortality was shown in the MSTO-211H lung mesothelioma cell line after treating first for 3 days with 10 mM citrate, a physiological inhibitor of PFK1 [52,53], and then with a low dose of cisplatin [54]. However, it should be noted from all of these studies, and to the best of the authors’ knowledge, that 2-DG, citrate, and cisplatin, each alone have never been shown to quickly eradicate real cancers in animals. This is in sharp contrast to 3BP, a novel, second-generation glycolysis inhibitor.

In a follow up study on rats, 3BP exhibited high anticancer activity toward quite large advanced cancers (hepatomas) [55]. In fact, every animal carrying an advanced hepatoma that was treated with 3BP became completely cancer free. Moreover, the cancer never recurred throughout the animals’ lifetime. About 70% of cancer cells were killed after one administration and after 4 weeks of treatment the tumors in all rats were eradicated without any harm to the animals. Thereafter, they all lived out a normal life without the return of cancer and died natural deaths. To the authors’ knowledge, in the long history of cancer research [56], no other anti-cancer agent has been shown to exhibit such striking, effective, rapid, remarkable, and unexpected results.

In a study published 4 years later [57], co-treatment with cisplatin and 3BP was shown to be much more efficient than treatment with cisplatin alone. This is probably due to the fact that 3BP causes ATP depletion leading to inactivation of the mechanism giving resistance to cisplatin, i.e., multidrug resistance phenotype (MDR) which is ATP-dependent. Therefore, 3BP is considered as a powerful multidrug resistance reversal modulator [58]. Considering its lack of evident side-effects [55], Ihrlund et al. [57] went on to remark that 3BP is a promising candidate for an anticancer therapeutic, both alone and in co-treatment with other drugs. This would include both animals and humans bearing cancers that exhibit positive PET scans.

### 2.3. Mechanism of Action of 3BP

3BP is a structural analog of pyruvic acid. It is highly reactive, showing strong alkylating properties toward some proteins/enzymes. The pyruvic chain preferentially and covalently binds to cysteine(s) of certain proteins, changing their conformation and activity [59]. Finally, the so-called protein pyruvylation results in alkylation of its active site and the release of a bromide radical [55]. Furthermore, 3BP and its derivatives have been considered as inhibitors of isocitrate lyase and malate synthase—key enzymes in the glyoxylate pathway that plays an important role in the pathogenesis of many fungal and bacterial infections, but does not exist in mammalian cells [60].

**Figure 1:**
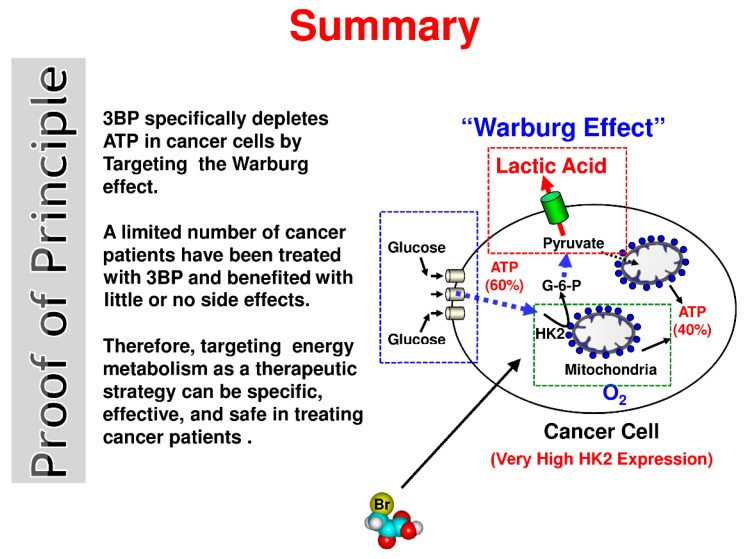
Summary, proof of principle—the simple mechanism by which 3-bromopyruvate (3BP) quickly and efficiently kills cancer cells through rapid energy depletion.

Although all details of the mechanism of action of 3BP toward mammalian cancer cells are probably not completely understood, based on all data collected to date, the authors believe that the simplest mechanism by which this “small molecule” quickly and efficiently kills cancer cells may be that which is depicted in the “Summary, Proof of Principle” (Figure 1). Here, a cancer cell is shown producing ATP, its energy source. As indicated in the text, in highly malignant cells, i.e., those that will likely kill nearly 1 of 2 men and 1 of 3 women, about 60% of the ATP is produced by glycolysis and the other 40% by the mitochondria [5]. In the cancer cell shown, the anticancer agent 3BP is envisioned to enter through MCTs, that are involved normally in the efflux of lactic acid out of the cell. It is very probable that in cancer cells, 3BP uptake is particularly effective because of the overexpression of MCTs [30]. The entry of 3BP is successfully achieved because lactic acid and 3BP differ in only a single atom (Br), making it impossible for the cancer cells’ MCTs to distinguish between the two.

Some researchers have questioned the important role of MCTs and suggest that a simple diffusion mechanism may also play a crucial role. Enhanced glycolysis observed inside cancer cells results in a pH gradient across the cell membrane. This in turn results in 3BP uptake specifically by cancer cells [61,62]. However, this role of the pH gradient established by tumor cells should not be overestimated. Thus, it has been suggested that this phenomenon enhances via MCTs-mediated transport [62] that could explain the specificity of 3BP’s effect on cancer cells. Then, once inside the cancer cell, 3BP inhibits its two energy (ATP) production factories, glycolysis and mitochondria. Normal cells, among them erythrocyte cells, are not harmed by the appropriate 3BP concentration, as they have a deficiency of MCTs [30,63]. HK2 bound to VDAC initiates cancer cell glycolysis and is likely one of the most important targets of 3BP as it is known to be inhibited by this agent [64]. Significantly, at the turn of this new century 3BP was shown to inhibit mitochondrial bound hexokinase [64], known from the earlier work noted above to be bound to VDAC [40] and to be the HK2 isoform [39]. The mitochondrial phosphate transporter, that is essential for ATP synthesis is also well known to be inhibited by compounds reacting with sulfhydryl groups (-SH) such as 3BP [65].

The second most important cellular target for 3BP is the GAPDH enzyme [33,66], that like HK2 is also important for glycolysis. Moreover, this enzyme is overexpressed in many human cancers and its growing intracellular level positively correlates with tumor progression [67,68].

Regardless of the cell model, 3BP as a very effective inhibitor of glycolysis as well as mitochondrial ATP synthesis rapidly depletes cellular ATP [55,69,70] and consequently leads to apoptosis [71]. After only 2 h induction in the presence of a defined concentration of 3BP, one can observe characteristic morphological changes of multiple myeloma (MM) cells indicating an advanced stage of apoptosis. The cells exhibit some specific changes in morphology characterized by the presence of so-called “apoptotic bodies” which surround the cells. The studies based on flow cytometry confirmed that the percentage of apoptotic cells increased with time [72]. Similar results were obtained in the case of 3BP’s effect on hepatocellular carcinoma cells [73,74], and human breast cancer cells [21,75].

The key to understanding the differences in sensitivity toward 3BP in the case of different eukaryotic cells is the transport of this compound, or more specifically, differences in the level of the kinetics of 3BP transport in different cell types as already shown [69,76]. For this reason, the overexpression of MCTs and/or a pH gradient existing across the cell membrane of cancer cells as a result of over activated glycolysis are the main causes of their high susceptibility to 3BP. This is in contrast to normal cells. Similar differences in 3BP uptake velocity and the level of accumulation of this compound inside the cells of taxonomically different fungi were observed earlier [69].

**Figure 2:**
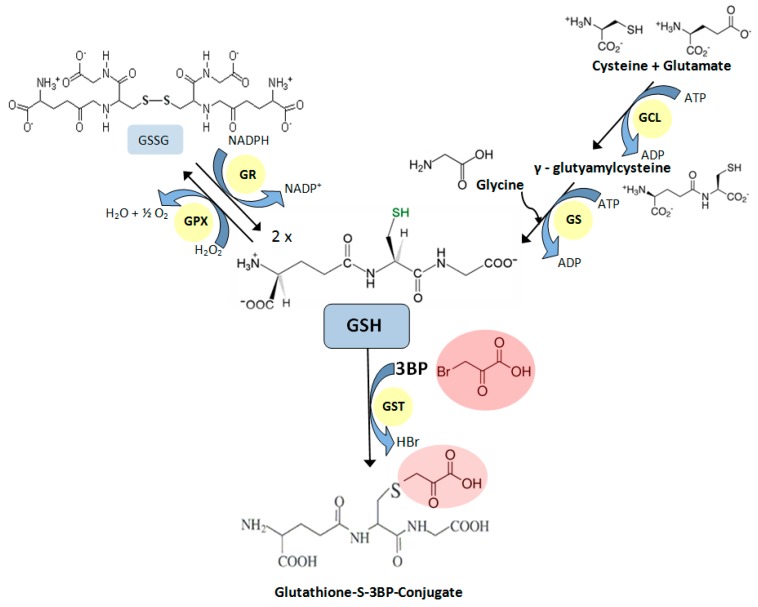
Glutathione metabolism. Crucial enzymes involved in glutathione metabolism: GCL—γ-glutamylcysteine synthetase; GS—glutathione synthetase; GR—glutathione reductase; GPX—glutathione peroxidase; GST—glutathione S-transferase.

Although the mechanism of action of 3BP is rather complex [70], it is well known that this compound generates ROS. Studies confirming this were carried out on MM [72], hepatoma [74], glioma [77], and breast cancer cells [75]. The accumulation of intracellular ROS occurs as the result of the inhibition of the mitochondrial respiratory chain [74,75]. One of the natural protection mechanisms of cells against the harmful effects of ROS is a reduced form of glutathione (GSH). For this reason, susceptibility of cells toward 3BP closely correlates with the natural, intracellular concentration of this tripeptide thiol [69,72,78]. This was also shown in studies on *Saccharomyces cerevisiae* mutants with deleted genes for the glutathione biosynthetic pathway [70]. Furthermore, recent studies show that 3BP causes a significant decrease in GSH concentration inside fungal, algal, and MM cells while maintaining the viability of cells similar to the control without 3BP [72]. This phenomenon occurs due to the GSH-3BP complex formation in order to inactivate this compound [79]. The enzyme that has the ability to catalyze the conjugation of the reduced form of glutathione to xenobiotic substrates is glutathione S-transferase (GST). The studies on *Cryptococcus neoformans* and MM cells demonstrated that under the influence of 3BP, the expression of the gene encoding this protein increases [72]. The overexpression of the GST genes leads to an enhancement of GSH-3BP complex formation. Moreover, it was shown that 3BP alters the level of the expression of genes encoding other crucial enzymes involved in GSH metabolism (Figure 2). Thus, the overexpression of genes encoding pivotal enzymes for the synthesis of GSH, γ-glutamylcysteine synthetase (GCL) and glutathione synthetase (GS) were also observed in response to the strong alkylating action of 3BP.

The increased concentration of GSH is conducive to enhanced inactivation of detrimental drugs. Accordingly, it is not surprising that 3BP demonstrates synergistic activity with other compounds that reduce intracellular levels of GSH. The examples of such compounds which act like GSH depletors are buthionine sulphoximine (BSO), methionine sulfoximine (MSO), and paracetamol (acetaminophen). BSO inhibits GCL, MSO irreversible restrains GS effectively blocks GSH synthesis, and paracetamol is metabolized to cytotoxic *N*-acetyl-4-benzoquinoneimine (NAPQI) that binds to the GSH [79,80,81,82,83]. Research performed on *Saccharomyces cerevisiae* and *Cryptococcus neoformans* confirmed that 3BP and BSO exhibit a strong synergistic effect [72,76,84]. Summarizing, what is crucial is that 3BP exhibits no obvious toxicity to healthy cells both in vitro in cell lines [57] and in vivo in animals [55,85]. Moreover, 3BP does not exhibit mutagenic properties in the Ames test [86]. As was shown in the yeast *S. cerevisiae* model, 3BP is not extruded by PDR (Pleiotropic Drug Resistance) efflux pumps, and it is unlikely that cancer cells would develop resistance via the MDR network [76].

Perhaps most significant is the fact that there is a human case report of 3BP usage as a therapeutic for primary liver cancer of the fibrolamellar carcinoma form (FLC) [73]. The patient’s doctors had exhausted commonly used treatment options and decided to use 3BP that had been shown to work well in an earlier study on hepatoma bearing rats [55]. After treatment of the patient with 3BP, a CT-guided puncture of ascites fluid showed lack of ascites tumor cells, suggesting that the tumors in the liver were well encapsulated and dead.

However, rapid destruction of the tumor cells resulted in the overloading of liver’s function. Almost a year after the beginning of treatment with 3BP and two years after the first diagnosis, the patient died because of an overload of liver function [73]. Nevertheless, the patient’s life had been extended enough to allow him to travel to the U.S. with his parents, participate as the patient in a filmed clinical correlation lecture at the Johns Hopkins University School of Medicine, Baltimore, MD, and thereafter to go on an extended vacation with his family prior to returning home to The Netherlands.

### 2.4. Perspectives

Cancer is the leading cause of death worldwide, accounting for 8.2 million deaths in 2012, according to the World Health Organization [87]. The chances of acquiring this disease are very high and it is predicted that one out of two men and one out of three women will die of cancer until the end of the 21st century [88]. The struggle to find an effective anticancer drug continues. Actually, one of the most extensively studied anticancer compounds is 3-bromopyruvate [89]. It is well known that 3BP enters cancer cells not only by specific MCTs but also by simple diffusion [61]. Therefore, the cell permeable ability of a drug is another crucial factor on which anti-cancer therapy [90] depends. For this reason, the pH gradient between the environment and the cancer cell cytoplasm may be crucial for more efficient diffusion, which should be enhanced with decreasing pH. Therefore, in therapy with 3BP some researchers suggest a hyperglycemia-inducing treatment [61]. In the context of considering more effective anticancer therapy with 3BP, ester derivatives of this compound should be mentioned. Among these, the most interesting seems to be 3-bromo-2-oxopropionate-1-propyl ester (3-BrOP). Such ester derivatives of 3BP may act as typical prodrugs which are considered by many researchers as more effective and safer therapies [90,91]. It is well known that a hydrophilic hydroxyl or carboxyl group on the starting compound can be converted to more lipophilic esters. Such prodrugs can be easily bio-converted to the biologically active drugs by universally present esterases throughout the human body [92,93]. Furthermore, the desired lipophilicity can be obtained by modification of the alkyl chain length. Such an effect was achieved in the case of a derivative of 3BP, i.e., 3-BrOP. This potent anticancer agent is more chemically stable than 3BP and similarly to the parent compound is also highly effective in inducing ATP depletion inside cancer cells [94]. However, it should be noted that we still do not know whether 3-BrOP penetrates the blood-brain barrier as effectively as 3BP [95].

Another interesting solution for more effective anticancer therapy could be a novel liposomal formulation of 3BP. This innovative form of the compound was evaluated for its permeability, inhibitory effects on HK2, and cytotoxic potential toward the human ovarian adenocarcinoma (SKOV-3) cells. According to recently published data, the liposomal formulation has better cell membrane permeability and inhibitory activity toward HK2 and also results in higher cytotoxicity toward the cancer cell line used in the study than the water solutions of 3BP [96]. Taking into account the two novel formulations of 3BP mentioned above, further research on the safety profile of these innovations is now needed.

In conclusion, it should be emphasized that it is not easy to find effective anticancer compounds. Ideally, the perfect anticancer agent should not only kill the primary tumor, but also any metastatic tumors and free tumor cells present in the blood [97]. Certainly, in this regard recent developments concerning the usage of 3BP in anti-cancer therapy provide encouragement. This is especially the case for those cancers that clearly exhibit positive PET scans/images, i.e., those with a robust “Warburg effect” dependent on mitochondrial bound HK2. Significantly, this includes almost all cancer types regardless of their tissue/organ of origin.

Finally, setting cancer aside, it is important to note that 3BP has been shown to also be a potent antifungal agent with very selective toxicity toward the human opportunistic pathogen *Cryptococcus neoformans* [69,84]. Cryptococcosis takes approximately 625,000 lives each year worldwide and on the African continent is even more frequent than tuberculosis [98]. Moreover, in vitro studies showed that 3BP could be successfully used in combination with atovaquone against *Toxoplasma gondii*. These studies also confirmed no effect of 3BP on host cell proliferation or viability [99].

### 2.5. Proper and Improper Use of 3BP

Finally, it should be emphasized that 3BP is a potential drug undergoing clinical development [73,100]. When properly formulated and used to treat either animals or humans, it has been shown to be a remarkably effective anti-cancer agent. As for any drug or other medication, 3BP must be properly formulated and never used in excess quantities. Unfortunately, very recently, and certainly to the authors surprise, a non-authorized, unprofessional, and dramatic application of 3BP by an individual with no knowledge about this small molecule and its proper formulation for use in animals or humans has been described by Hinnerk Feldwisch-Drentrup in Science [101]. For those skilled in the art, it is well known that drug toxicity is a question of purity (human grade) of the compound and is directly related to the ability to properly prepare a solution of suitable composition, concentration, formula, and pH. Please note that even the best drug used in high concentrations can be toxic due to side and secondary effects. Certainly, 3BP at the appropriate concentration and formulated with the correct solution choice is non-toxic to animals (mice, rats, rabbits, pigs) and humans, effectively destroys tumors that exhibit a Warburg effect, and is therefore detected using PET diagnostic technology. It is worth mentioning that presently in a few places in the world, including the Dayspring Cancer Clinic in Arizona (http://www.dayspringcancerclinic.com) effective clinical trials are being conducted for the treatment of various types of cancer using 3BP. Due to the forecasted “epidemic of cancer” in the coming years, it is hoped that this relatively inexpensive and effective anti-cancer and antifungal drug will pass successfully all clinical trials and will be available as soon as possible on the shelf at pharmacies throughout the world.

## References

[1] Warburg O., Wind F., Negelein E. (1927). The metabolism of tumors in the body. J. Gen. Physiol..

[2] Kennedy E.P., Lehninger A.L. (1948). Intracellular structures and the fatty acid oxidase system of rat liver. J. Biol. Chem..

[3] Lehninger A.L., Kennedy E.P. (1948). The requirement of the fatty acid oxidase complex of rat liver. J. Biol. Chem..

[4] Warburg O. (1956). On the origin of cancer cells. Science.

[5] Nakashima R.A., Paggi M.G., Pedersen P.L. (1984). Contributions of glycolysis and oxidative phosphorylation to adenosine 5-triphosphate production in AS-30D hepatoma cells. Cancer Res..

[6] Icard P., Poulain L., Lincet H. (2012). Understanding the central role of citrate in the metabolism of cancer cells. Biochim. Biophys. Acta.

[7] Diaz-Ruiz R., Rigoulet M., Devin A. (2011). The Warburg and Crabtree effects: On the origin of cancer cell energy metabolism and of yeast glucose repression. Biochim. Biophys. Acta.

[8] Pedersen P.L., Greenawalt J.W., Chan T.L., Morris H.P. (1970). A Comparison of Some Ultrastructural and Biochemical Properties of Mitochondria from Morris Hepatomas 9618A, 7800 and 3924A. Cancer Res..

[9] Pedersen P.L. (1978). Tumor mitochondria and the bioenergetics of cancer cells. Prog. Exp. Tumor Res..

[10] Weber G., Stubbs M., Morris H.P. (1971). Metabolism of hepatomas of different growth rates in situ and during ischemia. Cancer Res..

[11] Diaz-Ruiz R., Uribe-Carvajal S., Devin A., Rigoulet M. (2009). Tumor cell energy metabolism and its common features with yeast metabolism. Biochim. Biophys. Acta.

[12] Crabtree H.G. (1929). Observations on the carbohydrate metabolism of tumors. Biochem. J..

[13] Broach J.R. (2012). Nutritional control of growth and development in yeast. Genetics.

[14] Van Urk H., Voll W.S.L., Scheffers W.A., van Dijken J.P. (1990). Transient-state analysis of metabolic fluxes in Crabtree-positive and Crabtree-negative yeasts. Appl. Environ. Microbiol..

[15] Christofk H.R., Vander Heiden M.G., Harris M.H., Ramanathan A., Gerszten R.E., Wei R., Fleming M.D., Schreiber S.L., Cantley L.C. (2008). The M2 splice isoform of pyruvate kinase is important for cancer metabolism and tumor growth. Nature.

[16] Romero-Garcia S., Moreno-Altamirano M.M., Prado-Garcia H., Sanchez-Garcia F.J. (2016). Lactate contribution to the tumor microenvironment: Mechanisms, effects on immune cells and therapeutic relevance. Front. Immunol..

[17] Alfarouk K.O., Verduzco D., Rauch C., Muddathir A.K., Adil H.H.B., Elhassan G.O., Ibrahim M.E. (2014). Glycolysis, tumor metabolism, cancer growth and dissemination. A new pH-based etiopathogenic perspective and therapeutic approach to an old cancer question. Oncoscience.

[18] Rodríguez-Enriquez S., Juárez O., Rodríguez-Zavala J.S., Moreno-Sánchez R. (2001). Multisite control of the Crabtree effect in ascites hepatoma cells. Eur. J. Biochem..

[19] Marroquin L.D., Hynes J., Dykens J.A., Jamieson J.D., Will Y. (2007). Circumventing the crabtree effect: Replacing media glucose with galactose increases susceptibility of HepG2 cells to mitochondrial toxicants. Toxicol. Sci..

[20] Grüning N.M., Lehrach H., Ralser M. (2010). Regulatory crosstalk of the metabolic network. Trends Biochem. Sci..

[21] Liu Z., Zhang Y.Y., Zhang Q.W., Zhao S.R., Wu C.Z., Cheng X., Jiang C.C., Jiang Z.W., Liu H. (2014). 3-Bromopyruvate induces apoptosis in breast cancer cells by down regulating Mcl-1 through the PI3K/Akt signaling pathway. Anticancer Drugs.

[22] DeBerardinis R.J., Cheng T. (2010). Q’s next: The diverse functions of glutamine in metabolism, cell biology and cancer. Oncogene.

[23] Park J.H., Vithayathil S., Kumar S., Sung P.L., Dobrolecki L.E., Putluri V., Bhat V.B., Bhowmik S.K., Gupta V., Arora K. (2016). Fatty acid oxidation-driven Src links mitochondrial energy reprogramming and oncogenic properties in triple-negative breast cancer. Cell Rep..

[24] Bluemlein K., Grüning N.M., Feichtinger R.G., Lehrach H., Kofler B., Ralser M. (2011). No evidence for a shift in pyruvate kinase PKM1 to PKM2 expression during tumorigenesis. Oncotarget.

[25] Hitosugi T., Kang S., Vander Heiden M.G., Chung T.W., Elf S., Lythgoe K., Dong S., Lonial S., Wang X., Chen G.Z. (2009). Tyrosine phosphorylation inhibits PKM2 to promote the Warburg effect and tumor growth. Sci. Signal..

[26] Christofk H.R., Vander Heiden M.G., Wu N., Asara J.M., Cantley L.C. (2008). Pyruvate kinase M2 is a phosphotyrosine-binding protein. Nature.

[27] Mazurek S., Boschek C.B., Hugo F., Eigenbrodt E. (2005). Pyruvate kinase type M2 and its role in tumor growth and spreading. Semin. Cancer Biol..

[28] Gupta V., Wellen K.E., Mazurek S., Bamezai R.N. (2013). Pyruvate kinase M2: Regulatory circuits and potential for therapeutic intervention. Curr. Pharm. Des..

[29] Kim J.W., Tchernyshyov I., Semenza G.L., Dang C.V. (2006). HIF-1-mediated expression of pyruvate dehydrogenase kinase: A metabolic switch required for cellular adaptation to hypoxia. Cell Metab..

[30] Pinheiro C., Longatto-Filho A., Azevedo-Silva J., Casal M., Schmitt F.C., Baltazar F. (2012). Role of monocarboxylate transporters in human cancers: State of the art. J. Bioenerg. Biomembr..

[31] Doherty J.R., Cleveland J.L. (2013). Targeting lactate metabolism for cancer therapeutics. J. Clin. Investig..

[32] Feron O. (2009). Pyruvate into lactate and back: From the Warburg effect to symbiotic energy fuel exchange in cancer cells. Radiother. Oncol..

[33] Birsoy K., Wang T., Possemato R., Yilmaz O.H., Koch C.E., Chen W.W., Hutchins A.W., Gultekin Y., Peterson T.R., Carette J.E. (2013). MCT1-mediated transport of a toxic molecule is an effective strategy for targeting glycolytic tumors. Nat. Genet..

[34] Seyfried T.N., Flores R.E., Poff A.M., D’Agostino D.P. (2014). Cancer as a metabolic disease: Implications for novel therapeutics. Carcinogenesis.

[35] Macheda M.L., Rogers S., Best J.D. (2005). Molecular and cellular regulation of glucose transporter (GLUT) proteins in cancer. J. Cell. Physiol..

[36] Yamamoto T., Seino Y., Fukumoto H., Koh G., Yano H., Inagaki N., Yamada Y., Inoue K., Manabe T., Imura H. (1990). Over-expression of facilitative glucose transporter genes in human cancer. Biochem. Biophys. Res. Commun..

[37] Bustamante E., Pedersen P.L. (1977). High aerobic glycolysis of rat hepatoma cells in culture: Role of mitochondrial hexokinase. Proc. Natl. Acad. Sci. USA.

[38] Bustamante E., Morris H.P., Pedersen P.L. (1981). Energy metabolism of tumor cells: Requirement for a form of hexokinase with a propensity for mitochondrial binding. J. Biol. Chem..

[39] Nakashima R.A., Paggi M.G., Scott L.J., Pedersen P.L. (1988). Purification and characterization of a bindable form of mitochondrial bound hexokinase from the highly glycolytic AS-30D rat hepatoma cell line. Cancer Res..

[40] Nakashima R.A., Mangan P.S., Colombini M., Pedersen P.L. (1986). Hexokinase receptor complex in hepatoma mitochondria: Evidence from *N*,*N*’-dicyclohexylcarbodiimide-labelling studies for the involvement of the pore-forming protein VDAC. Biochemistry.

[41] Arora K.K., Pedersen P.L. (1988). Functional significance of mitochondrial bound hexokinase in tumor cell metabolism. Evidence for preferential phosphorylation of glucose by intra-mitochondrially generated ATP. J. Biol. Chem..

[42] Pedersen P.L. (2008). Voltage dependent anion channels (VDACs): A brief introduction with a focus on the outer mitochondrial compartment’s roles together with hexokinase-2 in the “Warburg effect” in cancer. J. Bioenerg. Biomembr..

[43] Mathupala S.P., Ko Y.H., Pedersen P.L. (2009). Hexokinase-2 bound to mitochondria: Cancer’s stygian link to the “Warburg Effect” and a pivotal target for effective therapy. Semin. Cancer Biol..

[44] Vora S., Halper J.P., Knowles D.M. (1985). Alterations in the activity and isozymic profile of human phosphofructokinase during malignant transformation in vivo and in vitro: Transformation- and progression-linked discriminants of malignancy. Cancer Res..

[45] Vander Heiden M.G., Cantley L.C., Thompson C.B. (2009). Understanding the Warburg effect: The metabolic requirements of cell proliferation. Science.

[46] Marín-Hernández A., Gallardo-Pérez J.C., Ralph S.J., Rodríguez-Enríquez S., Moreno-Sánchez R. (2009). HIF-1alpha modulates energy metabolism in cancer cells by inducing over-expression of specific glycolytic isoforms. Mini Rev. Med. Chem..

[47] Mathupala S.P., Rempel A., Pedersen P.L. (2001). Glucose catabolism in cancer cells: Identification and characterization of a marked activation response to hypoxic conditions. J. Biol. Chem..

[48] Robey R.B., Hay N. (2005). Mitochondrial hexokinases: Guardians of the mitochondria. Cell Cycle.

[49] Pastorino J.G., Hoek J.B. (2008). Regulation of hexokinase binding to VDAC. J. Bioenerg. Biomembr..

[50] Salk J.J., Fox E.J., Loeb L.A. (2010). Mutational heterogeneity in human cancers: Origin and consequences. Annu. Rev. Pathol..

[51] Zhang X.D., Deslandes E., Villedieu M., Poulain L., Duval M., Gauduchon P., Schwartz L., Icard P. (2006). Effect of 2-deoxy-D-glucose on various malignant cell lines in vitro. Anticancer Res..

[52] Lu Y., Zhang X., Zhang H., Lan J., Huang G., Varin E., Lincet H., Poulain L., Icard P. (2011). Citrate induces apoptotic cell death: A promising way to treat gastric carcinoma?. Anticancer Res..

[53] Icard P., Zhang X.D., Varin E., Allouche S., Coquerel A., Paciencia M., Joyeux L., Gauduchon P., Lincet H., Poulain L., Zubritsky A. (2012). Why Anti-Energetic Agents Such as Citrate or 3-Bromopyruvate should be Tested as Anti-Cancer Agents: Experimental In Vitro and In Vivo Studies. Mesotheliomas—Synonyms and Definition, Epidemiology, Etiology, Pathogenesis, Cyto-Histopathological Features, Clinic, Diagnosis, Treatment, Prognosis.

[54] Zhang X., Varin E., Allouche S., Lu Y., Poulain L., Icard P. (2009). Effect of citrate on malignant pleural mesothelioma cells: A synergistic effect with cisplatin. Anticancer Res..

[55] Ko Y.H., Smith B.L., Wang Y., Pomper M.G., Rini D.A., Torbenson M.S., Hullihen J., Pedersen P.L. (2004). Advanced cancers: Eradication in all cases using 3-bromopyruvate therapy to deplete ATP. Biochem. Biophys. Res. Commun..

[56] Sudhakar A. (2009). History of cancer, ancient and modern treatments. J. Cancer Sci. Ther..

[57] Ihrlund L.S., Hernlund E., Khan O., Shoshan M.C. (2008). 3-Bromopyruvate as inhibitor of tumour cell energy metabolism and chemopotentiator of platinum drugs. Mol. Oncol..

[58] Wu L., Xu J., Yuan W., Wu B., Wang H., Liu G., Wang X., Du J., Cai S. (2014). The Reversal Effects of 3-Bromopyruvate on Multidrug Resistance in Vitro and In Vivo Derived from Human Breast MCF-7/ADR Cells. PLoS ONE.

[59] Ko Y.H., McFadden B.A. (1990). Alkylation of isocitrate lyase from *Escherichia coli* by 3-bromopyruvate. Arch. Biochem. Biophys..

[60] Krátký M., Vinšová J. (2012). Advances in mycobacterial isocitrate lyase targeting and inhibitors. Curr. Med. Chem..

[61] Dell’Antone P. (2013). Enhancing 3-bromopyruvate toxicity in tumor cells by inducing hyperglycemia. Pharmacologia.

[62] Dell’Antone P. (2012). Energy metabolism in cancer cells: How to explain the Warburg and Crabtree effects?. Med. Hypotheses.

[63] Sadowska-Bartosz I., Soszyński M., Ułaszewski S., Ko Y.H., Bartosz G. (2014). Transport of 3-bromopyruvate across the human erythrocyte membrane. Cell Mol. Biol. Lett..

[64] Ko Y.H., Pedersen P.L., Geschwind J.F. (2001). Glucose catabolism in the rabbit VX2 tumor model for liver cancer: Characterization and targeting hexokinase. Cancer Lett..

[65] Kaplan R.S., Pratt R.D., Pedersen P.L. (1986). Purification and characterization of the reconstitutively active phosphate transporter from rat liver mitochondria. J. Biol. Chem..

[66] Cardaci S., Desideri E., Ciriolo M.R. (2012). Targeting aerobic glycolysis: 3-bromopyruvate as a promising anticancer drug. J. Bioenerg. Biomembr..

[67] Ramos D., Pellín-Carcelén A., Agustí J., Murgui A., Jordá E., Pellín A., Monteagudo C. (2015). Deregulation of glyceraldehyde-3-phosphate dehydrogenase expression during tumor progression of human cutaneous melanoma. Anticancer Res..

[68] Wang D., Moothart D.R., Lowy D.R., Qian X. (2013). The expression of glyceraldehyde-3-phosphate dehydrogenase associated cell cycle (GACC) genes correlates with cancer stage and poor survival in patients with solid tumors. PLoS ONE.

[69] Dyląg M., Lis P., Niedźwiecka K., Ko Y.H., Pedersen P.L., Goffeau A., Ułaszewski S. (2013). 3-Bromopyruvate: A novel antifungal agent against the human pathogen Cryptococcus neoformans. Biochem. Biophys. Res. Commun..

[70] Lis P., Jurkiewicz P., Cal-Bąkowska M., Ko Y.H., Pedersen P.L., Goffeau A., Ułaszewski S. (2016). Screening the yeast genome for energetic metabolism pathways involved in a phenotypic response to the anti-cancer agent 3-bromopyruvate. Oncotarget.

[71] Sun Y., Liu Z., Zou X., Lan Y., Sun X., Wang X., Zhao S., Jiang C., Liu H. (2015). Mechanisms underlying 3-bromopyruvate- induced cell death in colon cancer. J. Bioenerg. Biomembr..

[72] Niedźwiecka K., Dyląg M., Augustyniak D., Majkowska-Skrobek G., Cal-Bąkowska M., Ko Y.H., Pedersen P.L., Goffeau A., Ułaszewski S. (2016). Glutathione may have implications in the design of 3-bromopyruvate treatment protocols for both fungal and algal infections as well as multiple myeloma. Oncotarget.

[73] Ko Y.H., Verhoeven H.A., Lee M.J., Corbin D.J., Vogl T.J., Pedersen P.L. (2012). A translational study “case report” on the small molecule “energy blocker” 3-bromopyruvate (3BP) as a potent anticancer agent: From bench side to bedside. J. Bioenerg. Biomembr..

[74] Kim J.S., Ahn K.J., Kim J.A., Kim H.M., Lee J.D., Lee J.M., Kim S.J., Park J.H. (2008). Role of reactive oxygen species-mediated mitochondrial dysregulation in 3-bromopyruvate induced cell death in hepatoma cells: ROS-mediated cell death by 3-BrPA. J. Bioenerg. Biomembr..

[75] Zhang Q., Zhang Y., Zhang P., Chao Z., Xia F., Jiang C., Zhang X., Jiang Z., Liu H. (2014). Hexokinase II inhibitor, 3-BrPA induced autophagy by stimulating ROS formation in human breast cancer cells. Genes Cancer.

[76] Lis P., Zarzycki M., Ko Y.H., Casal M., Pedersen P.L., Goffeau A., Ułaszewski S. (2012). Transport and cytotoxicity of the anticancer drug 3-bromopyruvate in the yeast Saccharomyces cerevisiae. J. Bioenerg. Biomembr..

[77] El Sayed S.M., El-Magd R.M., Shishido Y., Yorita K., Chung S.P., Tran D.H., Sakai T., Watanabe H., Kagami S., Fukui K. (2012). D-Amino acid oxidase-induced oxidative stress, 3-bromopyruvate and citrate inhibit angiogenesis, exhibiting potent anticancer effects. J. Bioenerg. Biomembr..

[78] Sadowska-Bartosz I., Bartosz G. (2013). The effect of 3-bromopyruvic acid on human erythrocyte antioxidant defense system. Cell Biol. Int..

[79] Sadowska-Bartosz I., Szewczyk R., Jaremko L., Jaremko M., Bartosz G. (2016). Anticancer agent 3-bromopyruvic acid forms a conjugate with glutathione. Pharmacol. Rep..

[80] Reliene R., Schiestl R.H. (2006). Glutathione depletion by buthionine sulfoximine induces DNA deletions in mice. Carcinogenesis.

[81] Meister A. (1988). Glutathione metabolism and its selective modification. J. Biol. Chem..

[82] Wolchok J.D., Williams L., Pinto J.T., Fleisher M., Krown S.E., Hwu W.J., Livingston P.O., Chang C., Chapman P.B. (2003). Phase I trial of high dose paracetamol and carmustine in patients with metastatic melanoma. Melanoma Res..

[83] Hinson J.A., Roberts D.W., James L.P. (2010). Mechanisms of acetaminophen-induced liver necrosis. Handb. Exp. Pharmacol..

[84] Dyląg M., Lis P., Ko J.H., Pedersen P.L., Goffeau A., Ułaszewski S. (2015). Use of the Composition of 3-Bromopyruvate as a Second Application of a Medicament for the Treatment of Fungal Infections, PAT.219802. http://regserv.uprp.pl/register/application;jsessionid=84F17C141B9A6E93D3C97D4FA2EB3188?lng=en&tab=no_data&number=P.399978.

[85] Geschwind J.F., Georgiades C.S., Ko Y.H., Pedersen P.L. (2004). Recently elucidated energy catabolism pathways provide opportunities for novel treatments in hepatocellular carcinoma. Expert. Rev. Anticancer Ther..

[86] Majkowska-Skrobek G., Augustyniak D., Lis P., Bartkowiak A., Gonchar M., Ko Y.H., Pedersen P.L., Goffeau A., Ułaszewski S. (2014). Killing multiple myeloma cells with the small molecule 3-bromopyruvate: Implications for therapy. Anticancer Drugs.

[87] Forman D., Ferlay J., Stewart B.W., Wild C.P. (2014). The global and regional burden of cancer. World Cancer Report 2014.

[88] Pedersen P.L. (2012). 3-Bromopyruvate (3BP) a fast acting, promising, powerful, specific, and effective “small molecule” anti-cancer agent taken from labside to bedside: Introduction to a special issue. J. Bioenerg. Biomembr..

[89] Azevedo-Silva J., Queirós O., Baltazar F., Ułaszewski S., Goffeau A., Ko Y.H., Pedersen P.L., Preto A., Casal M. (2016). The anticancer agent 3-bromopyruvate: A simple but powerful molecule taken from the lab to the bedside. J. Bioenerg. Biomembr..

[90] Patrick G.L. (2013). An Introduction to Medicinal Chemistry.

[91] Yuan S., Wang F., Chen G., Zhang H., Feng L., Wang L., Colman H., Keating M.J., Li X., Xu R.H. (2013). Effective elimination of cancer stem cells by a novel drug combination strategy. Stem. Cells.

[92] Liederer B.M., Borchardt R.T. (2006). Enzymes involved in the bioconversion of ester-based prodrugs. J. Pharm. Sci..

[93] Taylor M.D. (1996). Improved passive oral drug delivery via prodrugs. Adv. Drug Deliv. Rev..

[94] Xu R.H., Pelicano H., Zhang H., Giles F.J., Keating M.J., Huang P. (2005). Synergistic effect of targeting mTOR by rapamycin and depleting ATP by inhibition of glycolysis in lymphoma and leukemia cells. Leukemia.

[95] Pierre K., Pellerin L. (2005). Monocarboxylate transporters in the central nervous system: Distribution, regulation and function. J. Neurochem..

[96] Gandham S.K., Talekar M., Singh A., Amiji M.M. (2015). Inhibition of hexokinase-2 with targeted liposomal 3-bromopyruvate in an ovarian tumor spheroid model of aerobic glycolysis. Int. J. Nanomed..

[97] Pedersen P.L. (2012). Mitochondria in relation to cancer metastasis: Introduction to a mini-review series. J. Bioenerg. Biomembr..

[98] Park B.J., Wannemuehler K.A., Marston B.J., Govender N., Pappas P.G., Chiller T.M. (2009). Estimation of the current global burden of cryptococcal meningitis among persons living with HIV/AIDS. AIDS.

[99] De Lima L.P.O., Seabra S.H., Carneiro H., Barbosa H.S. (2015). Effect of 3-Bromopyruvate and Atovaquone on Infection during In Vitro Interaction of *Toxoplasma gondii* and LLC-MK2 Cells. Antimicrob. Agents Chemother..

[100] PreScience Labs Announced that the FDA Accepts IND Application for Novel Oncology Drug. http://www.presciencelabs.com/prescience-labs-investors/press.php.

[101] Feldwisch-Drentrup H. (2016). Candidate cancer drug suspected after death of three patients at an alternative medicine clinic. Science.

